# Lung Ultrasound in Early Diagnosis of Neonatal Ventilator Associated Pneumonia before Any Radiographic or Laboratory Changes

**DOI:** 10.1155/2016/4168592

**Published:** 2016-11-06

**Authors:** Mohammed Ibrahim, Ahmed Omran, Mostafa Ibrahim, Nouran Bioumy, Sonya El-Sharkawy

**Affiliations:** ^1^Departments of Pediatrics & Neonatology, Faculty of Medicine, Suez Canal University, Ismailia, Egypt; ^2^Department of Radiodiagnosis, Faculty of Medicine, Suez Canal University, Ismailia, Egypt

## Abstract

Neonatal pneumonia is reported to be the primary cause of neonatal respiratory failure and one of the common causes of neonatal hospitalization and death in developing countries. Chest X-ray was considered the gold standard for diagnosis of neonatal pneumonia. Lung ultrasonography has been described as a valuable noninvasive tool for the diagnosis of many neonatal pulmonary diseases. We report a case of ventilation associated neonatal pneumonia with very early diagnosis using lung ultrasound before any significant radiographic changes in chest X-ray or laboratory findings suggestive of infection.

## 1. Introduction

Neonatal pneumonia is a serious respiratory infectious disease considered the most common infectious disease in neonates and is a significant cause of neonatal deaths. Worldwide neonatal pneumonia accounts for up to 10% of childhood mortality, with the highest case fatality rates being reported in the developing countries [1, 2].

In neonatal pneumonia timely and accurate diagnosis is important to start appropriate treatment early and improve the patient outcome. In the past, diagnosis of neonatal pneumonia was mainly depending on the clinical diagnosis and chest radiographic findings. Recently, lung ultrasound (LUS) emerged as a modality of diagnosis in multiple neonatal pulmonary disorders including respiratory distress syndrome (RDS) [3], meconium aspiration syndrome (MAS) [4], transient tachypnea of the newborn (TTN) [5], and neonatal pneumonia [6].

Here, we present a case of ventilator associated neonatal pneumonia (VAP) with very early diagnosis using LUS before either radiographic or laboratory changes.

## 2. Case Presentation

A one-day-old female newborn was admitted to the neonatal intensive care unit (NICU) of Suez Canal University, Ismailia, Egypt, after uneventful prolonged vaginal delivery ended with urgent cesarean section (CS). At admission the neonate suffered from depressed activity and grade IV respiratory distress with the need for mechanical ventilation.

The prenatal history was insignificant apart from premature rupture of membranes 12 hours before the delivery. There is no history of maternal urinary tract infection, asthma, or diabetes mellitus. The trial of the vaginal delivery lasted for 10 hours with failure to progress and fetal distress on the recording cardiotocography that urged CS delivery. The APGAR score at 1 minute was 2, at 5 minutes was 4, and at 10 min was 4.

**Figure 1:**
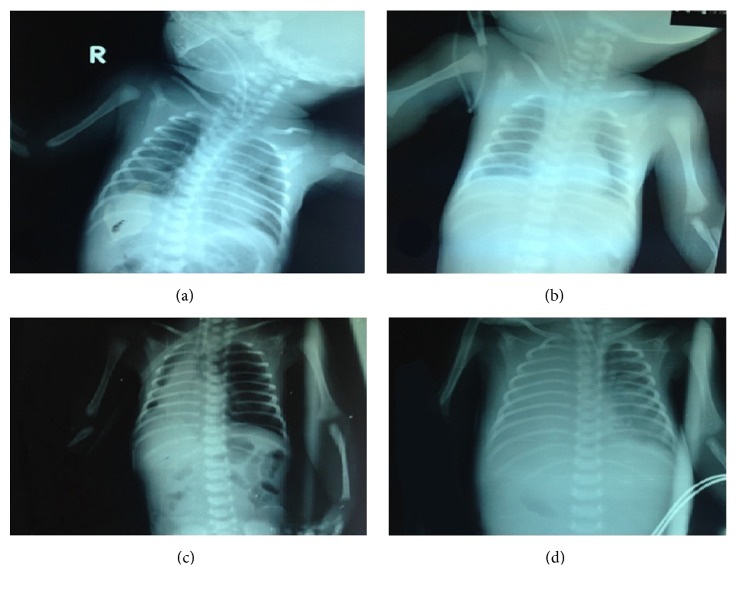
The serial radiographic changes with the development of VAP. A serial of plain chest radiographs along twelve days was obtained in supine anteroposterior position. The first five-day images (a and b) showed normal aeration with average lung volumes, normal mediastinum, and clear costophrenic angles. Also prominent bronchovascular markings are seen, but there is no focal pulmonary lesions. On 6-day image (c), an opacity is developed on the right lower lung zone with air-bronchogram and obliteration of the right costophrenic and cardiophrenic angles, suggesting consolidation. By the twelfth day (d), complete opacification of the right lung is seen causing opaque right hemithorax with mild tracheal deviation to the contralateral left side suggesting development of right pleural effusion. Also small consolidation patches are seen in the left para-cardiac region in middle and lower left lung zones with obliteration of the left cardiac border.

**Figure 2:**
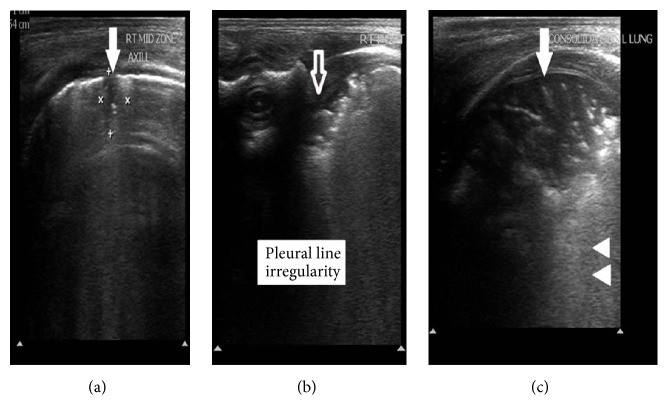
The LUS findings associated with VAP. Superficial high resolution lung ultrasound was done and images obtained in midaxillary (a), scapular (b), and midclavicular (c) lines at transverse and longitudinal planes. Multiple peripheral hypoechoic areas are seen which assume subpleural location showing irregular borders and air-bronchogram inside, suggesting pneumonic consolidations (solid arrows). Also thickened irregular pleural borders (empty arrow) with absent A-lines and coalescent B-lines around the consolidations (arrow heads).

By the end of the 2nd day the basic laboratory investigations were withdrawn showing no abnormal findings with C-reactive protein (CRP) value 2 mg/dL. The chest X-ray (CXR) showed only increased bronchovascular markings (Figure 1(a)). On the next day the Glasgow Coma Scale (GCS) of the patient started to improve with progressive lowering the settings of the mechanical ventilator. LUS screening was done after the arterial blood gases (ABGs) showed impaired oxygenation and ventilation and the new CXR was free (Figure 1(b)). The LUS showed multiple large consolidations with thickened irregular pleural line, absent A-lines, and coalescent B-lines around the consolidations suggesting the possibility of ventilator associated pneumonia (VAP) (Figures 2(a), 2(b), and 2(c)). A new CRP at this point also was negative (3 mg/dL) with leucocytic count 5,600/cmm and negative cultures.

One day later the settings of the mechanical ventilator became higher, and the follow-up CXR showed right lower pneumonic consolidation associated with collapse. Lung auscultation did not reveal any fine or coarse crepitations but only slightly diminished air entry over the right side. The CRP started to rise a day later (56 mg/dL) with increased leucocytic count (18,600/cmm) with absolute neutrophilia. Three days later the blood culture was positive. The diagnosis of VAP was made and the antibiotics were changed according to the culture and sensitivity results.

The 7th day witnessed deterioration of the general condition with markedly impaired aeration of the right lung. A new plain CXR was done (Figure 1(c)) showing large opacity developed on the right lower lung zone with air-bronchogram and obliteration of the right costophrenic and cardiophrenic angles, suggesting consolidation collapse.

The following 3 days showed no improvement in either the chest condition or the ventilator settings guided by the ABGs. So the antibiotics were changed to Vancomycin, Imipenem/Cilastatin, and Diflucan according to the local NICU protocols of antibiotics' usage.

By the 11th day, there was progressive deterioration with inadequate response to the medical and ventilatory support. A new plain CXR (Figure 1(d)) which showed complete opacification of the right lung is seen causing opaque right hemithorax. Also small consolidation patches are seen in the left para-cardiac region in middle and lower left lung zones with obliteration of the left cardiac border.

The failure of ventilation and oxygenation on high ventilator settings showed the need to transfer the neonate to a center with high frequency oscillatory ventilation (HFOV) or extracorporal membrane oxygenation (ECMO). However, there was no available place for transfer at that time. Respiratory failure then developed with eventual cardiac arrest.

## 3. Discussion

Respiratory disorders are the most frequent causes of admission to the NICU in both term and preterm infants. Worldwide pneumonia accounts for 15% of the total number of deaths in children less than 5 years [7]. Early diagnosis of neonatal pneumonia still remains a diagnostic challenge [8]. Using X-ray as a “gold standard” for the diagnosis of pneumonia, however, it should be used cautiously in neonates because of the potential late adverse effects of ionized radiation [9, 10], and the lack of findings on chest radiographs does not rule out the diagnosis if there is a strong suspicion of pneumonia.

Recently, there has been a great interest in developing new tools to increase the accuracy of pneumonia diagnosis with simultaneous decrease in exposure to ionized radiation especially in neonates. Advances in ultrasound technology have made LUS a potential attractive option for the diagnosis of pneumonia. Moreover, ultrasound is safe, portable, inexpensive, and relatively easy to teach.

A recent meta-analysis of studies comparing lung ultrasound versus CXR and chest CT scan for the diagnosis of pneumonia showed good sensitivity and specificity in children and adults [11, 12]. LUS was very close to CXR in identifying pulmonary abnormalities among children with suspected pneumonia with a 97% specificity for CXR-confirmed pneumonia [13]. Similarly, LUS had an overall sensitivity of 86% and specificity of 89% for diagnosing pneumonia when compared with CXR as reference standard [14].

Current evidence supports LUS as an imaging alternative for the diagnosis of neonatal respiratory disorders. Liu et al.'s study demonstrates that LUS is useful for the diagnosis of sever neonatal pneumonia [6]. LUS is also a reliable method to diagnose RDS and TTN in newborns with respiratory distress, with high sensitivity and specificity comparable with CXR [3]. LUS is a useful and promising tool in the diagnosis and management of MAS [4]. LUS is an accurate and reliable method for diagnosing neonatal pulmonary atelectasis (NPA) [15]. LUS is found to be superior to CXR in the detection of complications of RDS, particularly consolidation, atelectasis, and microabscesses [16].

The LUS in our case showed very early signs of pneumonia including multiple large consolidations with thickened pleural line and absent A-lines. Liu et al. reported that large areas of lung consolidation with irregular margins had 100% sensitivity and 100% specificity for the diagnosis of neonatal pneumonia; they also reported pleural line abnormalities in 90% of their cases [6].

In our reported case LUS detected signs of pneumonia before any change in CXR and also before any change in the laboratory markers. In cases of neonatal pulmonary atelectasis, LUS was more sensitive in detecting occult lesions than CXR [15].

In this case report we present an early and accurate diagnosis of VAP using LUS in Egyptian neonate before any radiographic or laboratory changes. This case may add a new dimension in using LUS as noninvasive method of diagnosis in neonatal VAP.
